# Associations among Substance Use, Mental Health Disorders, and Self-Harm in a Prison Population: Examining Group Risk for Suicide Attempt

**DOI:** 10.3390/ijerph14030317

**Published:** 2017-03-20

**Authors:** Madison L. Gates, Asher Turney, Elizabeth Ferguson, Veronica Walker, Michelle Staples-Horne

**Affiliations:** 1 Department of Family Medicine, Medical College of Georgia, Institute of Public and Preventive Health, Augusta University, 1120 15th Street, CJ 2300, Augusta, GA 30912, USA; 2 Centurion, LLC, 53 Century Blvd, Suite 150, Nashville, TN 37214, USA; aturney@centuriontn.com; 3 Department of Psychiatry and Health Behavior, Medical College of Georgia, Augusta University, 997 St. Sebastian Way, Augusta, GA 30912, USA; elizabeth@frmrisk.com; 4 Lexington Public Library, 3628 Walden Drive, Lexington, KY 40517, USA; vwalker@lexpublib.org; 5 Georgia Department of Juvenile Justice, Central Office, 3408 Covington Highway, Decatur, GA 30032, USA; michellestaples-horne@djj.state.ga.us

**Keywords:** mental health, prisoners, public health, self-injurious behavior, substance-related disorders, suicide, vulnerable population

## Abstract

Substance use disorders (SUD) and mental health disorders are significant public health issues that co-occur and are associated with high risk for suicide attempts. SUD and mental health disorders are more prevalent among offenders (i.e., prisoners or inmates) than the non-imprisoned population, raising concerns about the risk of self-harm. This cross-sectional study examined the population of a state prison system (10,988 out of 13,079) to identify associations among SUD (alcohol, cannabis, intravenous drugs, narcotics, and tobacco smoking), mental health disorders (anxiety, bipolar, depression, and psychotic disorders), and suicide attempts. The primary aim was to determine which groups (SUD, mental health disorders, and co-occurrences) were strongly association with suicide attempts. Groups with a documented SUD or mental health disorders compared to peers without these issues had 2.0 and 9.2 greater odds, respectively, for attempting suicide, which was significant at *p* < 0.0001 for both conditions. There were also significant differences within SUD and mental health disorders groups in regard to suicide attempts. Groups with the greatest odds for suicide attempts were offenders with comorbid bipolar comorbid and anxiety, alcohol combined with depression, and cannabis co-occurring with depression. Documentation of suicide attempts during imprisonment indicates awareness, but also suggest a need to continue enhancing screening and evaluating environmental settings.

## 1. Introduction

Substance use disorders (SUD) and mental health disorders are significant public health issues that often co-occur and are associated with functional disability [1] and high risk for self-harm, including suicide attempts and completion [2,3,4,5,6,7,8]. The prevalence for SUD and mental health disorders are much greater for the offender population (i.e., prisoners, inmates) than non-imprisoned populations [9,10,11]. Individuals with SUD have a significantly higher probability of being arrested compared to the population without these issues [12,13]. A majority of offenders in the United States (56%) reported that they used at least one substance prior to arrest [14]. Cannabis use was reported by 40% of offenders, followed by 21.4% for cocaine or crack, 12.2% for methamphetamine and amphetamine, and 8.2% for heroin/opioids [14], compared to 3.0% of non-imprisoned adults who indicated taking any substance. Furthermore, offenders who used cannabis, cocaine, methamphetamine, or heroin reported that they used these substances regularly [14].

The risk for criminal justice involvement increases when SUD and mental health disorder co-occur [13]. The United States criminal justice system has filled the void created by insufficient mental health services and has become a provider of last resort for some individuals with mental health disorders [15]. A large percent of offenders (24%) in state prison systems reported having recent mental health problems and 49% had symptoms for mental health disorders [16]. The Bureau of Justice Statistics found that 43.2% of the offender population had symptoms for mania disorder, followed by major depressive (23.5%) and psychotic disorder (15.4%) [16], which compares to non-imprisoned population estimates of 0.4%–2.1% for mania (one study reported 6.4% as an upper estimate), 6.0%–7.9% for major depression, and 3.1% for psychoses [3,16,17,18,19]. A large percent of offenders (41.7%) also have symptoms or diagnosed mental health disorders that co-occur with a SUD (e.g., alcohol, cannabis, cocaine, heroin, tobacco smoking) [16].

There is a strong relationship between mental health disorders and self-harm (e.g., suicide ideation, attempts, and other self-injurious behaviors) [20,21,22,23,24,25,26,27,28]. In particular, depression and bipolar with anxiety disorders are strongly associated with suicidal ideation, attempts, and completion [3,20,21,22,23,24,26,29,30,31]. Individuals with SUD also have greater incidence of suicidal ideation and attempts compared to the population who do not have a problem with substances [32,33]. Co-occurrence of SUD and mental health disorders increases the risk of suicidal ideation, attempts, and completion [2,3,4,5,6,7,27,28,33]. Cannabis, alcohol, and nicotine (tobacco smoking) use disorders that co-occur with mental health disorders significantly increase the risk of suicides [4,5,29,34,35,36].

Offenders greatly exceed the non-imprisoned population in regard to mental health disorders and SUD, particularly cannabis. In addition to a greater prevalence of risk factors, such as SUD and mental health disorders, suicide attempts among offenders exceed the percent for non-imprisoned populations [37,38]. Estimates of suicide attempts among offenders are 2.3% compared to 0.4% among non-imprisoned populations [38]. Among offenders who have depressive or manic symptoms, 13% reported attempting suicide [16]. Despite offenders having greater risk factors for suicide attempts (e.g., SUD and mental health disorders) compared to non-imprisoned populations, there are few investigations that have included this group. This descriptive cross-sectional study of a state department of corrections investigated the association among SUD, mental health disorders, and suicide attempts.

The primary aim of this study was to examine risk factors related to suicide attempts in an offender population. We hypothesized (1) there will be sociodemographic differences (gender, race, education, and security level) in regard to suicide attempts, (2) group proportions for suicide attempts will be greater for the population with co-occurring SUD and mental health disorders compared to offenders with either a SUD or a mental illness, and (3) the population with the conditions of bipolar and anxiety disorders and co-occurring cannabis use disorder will have the greatest odds of suicide attempts compared to other SUD that co-occur with other mental health disorders.

## 2. Materials and Methods

This cross-sectional descriptive study was approved by an institutional review (IRB) at an academic health center and was conducted in collaboration with a department of corrections (DOC) in the east south central region of the United States. The IRB protocol number is 10-0382F2L.

### 2.1. Inclusion and Exclusion Criteria

All offenders (men and women) imprisoned between 1 June 2005 and 31 December 2010 and had a record in the electronic health and offender management systems were included. Inclusion criteria also included all race and ethnic groups, and offenders who had date of birth, which was used to calculate age, all of which may be explanatory factors for suicide attempts. This DOC had thirteen facilities, of which two were women only and eleven men only. Facilities were dispersed geographically throughout the state, representing the geographic distribution of the state.

### 2.2. Data Collection

The data sources for this study were an electronic health record (EHR) and an offender management system (OMS), which the DOC we collaborated with used to manage offender health and to track and monitor their activities. All offenders receive a complete physical, mental, behavioral health, and dental examination upon arrival at the DOC reception center, also referred to as the intake facility. Health findings are recorded in the EHR by clinic staff (e.g., physicians, advanced registered nurse practitioners, nurses, and medical records specialists). The EHR is comprised of structured data (i.e., clinic staff entered data using standardized data dictionaries). Clinic staff entered diagnoses using the International Statistical Classification of Diseases (ICD-9) and the Systematized Nomenclature of Medicine (SNOMED) at the reception center and throughout offenders’ imprisonment as health conditions are identified and change. Documentation with ICD-9 provides a consistent and internationally recognized way to refer to diseases and disorders. This study extracted all health data (i.e., SUD, diagnoses for mental health disorders, and suicide attempts) from the EHR (See Table 1). Documentation of SUD were made on the basis of screening tools and limitedly on health records prior to imprisonment that offenders provided. Mental health disorders were diagnosed after extensive evaluation by psychologists and psychiatrists. Diagnoses for panic, phobia, and posttraumatic stress syndrome were included with anxiety disorders, as other investigators have grouped these conditions together [19,24,31,39]. Clinic staff documented suicide attempts during imprisonment based on physical evidence and investigations conducted by medical, mental health staff, and correctional officers. The OMS was created electronically from court records and managed by correctional staff (e.g., officers and case managers) throughout offenders’ imprisonment. This study extracted sociodemographic, criminal offense, security level, sentence, earliest potential parole, and release dates from the OMS.

### 2.3. Statistical Analyses

We used SAS^®^ 9.4 (SAS Institute, Cary, NC, USA) to conduct all statistical tests. Frequencies and percentages were conducted for race, gender, education, security level, SUD, and mental health disorders (i.e., nominal and categorical variables). Race comparisons were made between African Americans and Whites; there were too few observations for other racial and ethnic groups for meaningful analyses. Means and standard deviations (SD) were calculated for age and date of diagnosis (continuous variables). Differences in population proportions and odds (i.e., offenders with SUD and mental health disorders who attempted suicide) were evaluated using chi-square (χ^2^). We created mutually exclusive groups for SUD (alcohol, cannabis, intravenous drugs, narcotics, and tobacco smoking) and mental health disorders (anxiety, bipolar, depression or depressive symptoms, and psychotic disorders) to minimize potential statistical noise that polysubstance and comorbid conditions may have on odds for suicide attempts. We created a group for co-occurrence of bipolar and anxiety disorders, since these conditions are highly comorbid and are associated with increased risk for suicide attempts [2,23,27,39,40,41]. Comparisons for suicide attempts in regard to age and duration of imprisonment were made using the Wilcoxon rank sum test. When examining multiple groups, we performed the Fisher exact test and made *p*-value adjustments (including Bonferroni) to evaluate significance of pairwise comparisons. The *p*-value adjustment identified which comparisons were significant and decreased the probability of Type I error (false positives).

## 3. Results

There were 10,988 out of 13,079 offenders (84%) who had valid records in the EHR and OMS (i.e., inclusion criteria for the study). The population was majority White and men (See Table 2) with a mean age of 37.5 (SD = 11.5), 95% CI (37.3, 37.7). The mean age for offenders with documented suicide attempts was 36.6 (SD = 9.6), 95% CI (35.6, 37.6) compared to 37.5 (11.6), 95% CI (37.3, 37.7) who did not attempt suicide. A plurality of the population had completed high school, had a primary offense related to property crimes (e.g., destruction of property, burglary, receiving stolen property), and were classified as medium security, which allows some freedom of movement within a facility, but under greater supervision and restriction than offenders whose status was minimum level (See Table 2). Education was self-reported and a large percent of offenders refused to report this information. We also did not have access to all criminal offense and security level information for all offenders, which resulted in approximately one-third to almost forty percent of these data being unavailable.

### 3.1. Prevalence of Substance Use Disorders, Mental Health Disorders, and Suicide Attempt

Substance use disorders were prevalent, in which 6629 offenders (60.3%) had at least one documented issue and 2205 (20.1%) had more than one problem. Tobacco smoking was the most frequently used substance, followed by cannabis and alcohol (See Table 3). A large percent of the population (28.0%) had a diagnosed mental health disorder (i.e., anxiety, bipolar, depression, and psychotic disorders), and 3.7% of offenders had comorbid conditions. Depression was the most frequently diagnosed mental health disorder, and bipolar disorders the least (See Table 3). Suicide attempts occurred in 3.4% of the population (See Table 3).

An overwhelming majority of the diagnoses for mental health disorders (92.8%) were made during imprisonment (See Table 3). The mean duration for diagnosing an offender with an anxiety diagnosis was 4.5 years (SD = 6.3), 95% CI (4.1, 4.9); 3.6 years (SD = 5.0), 95% CI (3.0, 4.1) for a bipolar disorder; 4.1 years (SD = 6.1), 95% CI (3.8, 4.4) for depression; and, 4.6 years (6.5), 95% CI (4.0, 5.2) for a psychotic disorder. Most suicide attempts also occurred during imprisonment (See Table 3). The mean number of years imprisoned prior to a suicide attempt was 5.2 years (6.6), 95% CI (4.4, 6.0).

### 3.2. Group Differences for Suicide Attempt

Whites, men, and the population with a SUD or mental health disorder had greater odds of suicide attempts compared to peers (See Table 4). A significantly larger percent of offenders classified as maximum security attempted suicide compared to groups with minimum or medium security levels (See Table 4). We conducted the Fisher’s exact test for analyses of variables with more than two groups (i.e., education and security level). A pairwise comparison among security levels indicated that all confinements significantly differed from one another after a *p*-value adjustment (Bonferroni). The odds for suicide attempts were significantly greater for the maximum level compared to minimum (*p* < 0.0001) and medium (*p* = 0.002), and a classification of medium was associated with an increased likelihood for knowingly engaging in behaviors that could be life ending compared to the minimum level (*p* < 0.0001). Although offenders with post-secondary education (some college, undergraduate, and graduate degrees) had a smaller percent for suicide attempts compared to groups with less educational attainment, the differences were not significant (See Table 4).

We created mutually exclusive groups (i.e., offenders who only have one condition) for all SUD and mental health disorders. Some SUD and mental health disorders were associated with increased risk for suicide attempts. There were significant group differences between smokers and non-smokers, as well as the population with a history of intravenous drug use (IDU) compared to those who did not inject drugs. Offenders with a history of IDU had greater odds for suicide attempts compared to their non-IDU peers and smokers compared to non-smokers were more likely to have a documented case of suicidal behaviors, i.e., tries (See Table 5). Offenders with alcohol, cannabis, or narcotic use disorders were not significantly different from peers who did not have an issue with these substances. Offenders with bipolar disorders had significantly greater odds for suicide attempts compared to the population without this mental health problem (See Table 5). Comparisons between offenders with or without depression and the group with or without dual diagnoses of bipolar and anxiety disorders indicated that the population with these mental health disorders were associated with greater odds for suicide attempts (See Table 5). There were no significant differences for groups with or without anxiety or psychotic disorders (See Table 5).

There were significant within group differences for suicide attempts in regard to SUD and mental health disorders (See Table 6). Offenders with a history of IDU or tobacco smoking had larger percentages for suicide attempts (See Table 6). A pairwise comparison, using Fisher’s exact test and adjusted *p*-values, indicated that SUD significantly differed from one another (See Table 7). Offenders who had issues with IDU or tobacco smoking significantly differed from the group that had no SUD history (See Table 7), i.e., a larger percent of individuals with IDU and tobacco smoking issues had instances of suicide attempts compared to the group with no SUD. Only alcohol and cannabis use disorders groups differed significantly from IDU and tobacco smoking (See Table 7). A smaller proportion of the groups with alcohol and cannabis use disorders attempted suicide compared to offenders with histories of IDU or tobacco smoking (See Table 7). However, there were no significant differences detected for suicide attempts when the group without a substance use disorder was compared to offenders who had issues with alcohol, cannabis, or narcotics (See Table 7).

In regard to mental health disorders, offenders with bipolar and anxiety had the largest percent of population for suicide attempts (See Table 6). A pairwise comparison for mental health disorders indicated that a significantly smaller percent of the population without a diagnosis had attempted to end their life compared to groups with either anxiety, bipolar, depression, psychoses, or comorbidities (i.e., bipolar and anxiety), see Table 7. The group with bipolar and anxiety disorders had a significantly larger percent of population with suicide attempts compared to groups with anxiety only, depression, or psychotic disorders (Table 7).

### 3.3. Odds of Suicide Attempt for Substance Use Disorder and Co-Occurring Mental Health Disorder

There were a few significant population differences while controlling for SUD. The population size for this study was not sufficient to conduct analyses for all mental health disorders while controlling for an SUD. The population with depression and the co-occurrence of alcohol, cannabis, or tobacco smoking had greater odds for suicide attempts compared to the groups that had these SUD, but had not been diagnosed as having a mental health disorder (See Table 8). Although alcohol and cannabis use disorders independently were not associated with suicide attempts, their co-occurrence with depression increased the odds of trying to end one’s life (See Table 8). Unlike alcohol and cannabis, tobacco smoking did not increase odds for attempting suicide (See Table 8).

## 4. Discussion

### 4.1. Prevalence for Corrections and Non-Imprisoned Populations

Substance use disorders and mental health disorders for this DOC are much more prevalent than the non-imprisoned population. National survey data for alcohol use disorder indicated that 3.5% of adults 18 or older in 2010 had this condition compared to 18.5% in this DOC [42]. Percentages for cannabis (17.4%), cocaine (7.8%), and heroin (0.5%) also exceeded 2010 national averages (1.0%, 0.3%, and 0.1%, respectively) [42]. Further, 35% of offenders in this DOC smoked tobacco compared to 19.3% in the non-imprisoned population [43]. In regard to mental health disorders, the prevalence of depression for this DOC was greater than the 2012 non-imprisoned population (17.1% compared to 7.9%) [19]. The population for this DOC also exceeded the 2012 non-imprisoned population percentages for bipolar (4.9% compared to 2.1%) [3] and psychotic disorders (6.2% compared to 3.1%) [19]. However, diagnoses for anxiety disorders in this DOC were slightly less than the 2005 non-imprisoned population, which included the same conditions as this study (16.4% compared to 18.1%) [44]. The percent of suicide attempts in our population of offenders were 3.4%, which compared to 0.4% for attempts in a national survey of a non-imprisoned population [45] and an estimate of 2.3% across prison systems in the United States [38].

### 4.2. Group Risk for Suicide Attempts

Whites compared to African Americans had significantly greater odds for suicide attempts [46,47], which is consistent with other investigations. Men had greater odds for attempting suicide compared to women, which was unexpected. Investigations with non-imprisoned individuals have found that men are less likely to attempt suicide [48,49], but have greater odds for completing suicide compared to women [49]. This finding is surprising, since women in this DOC were significantly more likely to have a mental health disorders, *p* < 0.0001, OR = 1.4, 95% CI [1.3, 1.6], which are known risk factors for suicide attempts. However, the lower odds for women attempting suicide may be explained by their greater likelihood for utilizing mental health services than men [50]. There were no significant difference (*p* = 0.12) in suicide attempts in regard to age, which did not coincide with investigations with non-offenders [20,27,29,41,51]. The population that smoked tobacco, as well as the group with bipolar, depression, or bipolar with anxiety disorder, had significantly greater odds for attempting suicide compared to offenders who did not smoke or have these mental health disorders, which investigations of non-imprisoned populations have found [8,33,52,53,54].

Surprisingly, we did not find increased odds for suicide attempts for groups that solely had an alcohol [4,6], cannabis [7,32,34], or narcotic use disorder [5]. Further, the population that was solely diagnosed with either anxiety [24,31,55] or a psychotic disorder [29,56] did not have greater odds for suicide attempts compared to peers without these mental health disorders. Although alcohol and cannabis were not associated independently with suicide attempts their co-occurrence with depression resulted in significantly greater odds for trying to commit suicide. The odds for suicide attempts for tobacco smokers who had anxiety or bipolar disorder was not significant, which is not consistent with other investigations [35,36,57,58,59].

Tobacco smokers who were diagnosed with depression did not have greater odds for suicide attempts compared to the population with depression only. While alcohol and cannabis use disorders co-occurring with depression were significantly associated with suicide attempts, we were unable to analyze the strength of the associations. However, neither cannabis use disorder nor anxiety independently resulted in greater odds for suicide attempts. Further, cannabis use disorder co-occurring with depression resulted in increased odds for suicide attempts compared to having only a mental health disorder. These findings suggest that past alcohol and cannabis use disorders may be important in regard to suicide attempts, but the mechanism that the substance may have is beyond the scope and aims of this investigation.

### 4.3. Unique Risk Factors for Offenders

The findings that maximum security was associated with increased odds for suicide attempts is not well defined, since there are few investigations that have included offenders. However, the increased odds for maximum security was not unexpected, since this security classification is the most restrictive level of imprisonment and is reserved for the most serious offenses, including serious disciplinary charges once imprisoned. In many correctional settings, including the department of corrections that was the source for our data, offenders classified at the maximum level reside in single bed units (i.e., they do not share living quarters), and contact with other offenders is minimal. The association between maximum security and suicide attempts may be related to offenders experiencing a negative environment, increased stress, reinforcement of negative self-worth, and social isolation [28,30,41]. The majority of offenders classified as maximum security were tobacco smokers (64.5%) and a plurality had a diagnosis of depression (38.4), significant risk factors for suicide attempts. Findings from this investigation suggest that greater odds for suicide attempts for offenders with co-occurring SUD and mental health disorders may differ somewhat from non-imprisoned populations and that moderating variables, such as gender and living environment, may have different influences on attempting suicide.

Although the aims of this investigation sought to identify associations among sociodemographics, SUD, mental health disorders, and suicide attempts, the strong links among these factors may provide corrections guidance in regard to assessing suicide risk and targeting resources to the population with the greatest odds of attempting to end their life. Tobacco smoking and the population with co-occurring bipolar and anxiety appear to have the greatest odds among SUD and mental health disorders respectively. The population with co-occurring SUD and mental health disorders, particularly tobacco smoking, cannabis, and alcohol use disorders, also had greater odds for suicide attempts. These characteristics are likely the initial risk factors for building a corrections-specific assessment for suicide attempts. Further, security classification, particularly maximum confinements, suggests that the environment of the level may need to be evaluated to identify how corrections can maintain security and safety, while also minimizing risk factors for suicide attempts, such as increased stress and social isolation.

The correctional population is a unique environment from non-imprisoned settings, such as security levels, overrepresentation of SUD, mental health disorders, and co-occurrences, limitations of agency to make decisions, large percent for less than a high school graduation and high prevalence of socially and economically disadvantaged backgrounds. Offenders also are separated involuntarily from their family (spouses, significant others, offspring), which may be stressors related to suicide attempts [33]. Despite these differences, suicide attempts is a major issue for the population with SUD and mental health disorders. This descriptive study provides preliminary results regarding the effect that moderating (e.g., race, gender, age, education, security level) and potential mediating variables (e.g., SUD, mental health disorders, co-occurrences) may have on suicide attempts in an offender population.

### 4.4. Limitations

This study is among a few investigations that have included an offender population to access the associations of SUD, mental health disorder, and self-harm (i.e., suicide attempts). Although the results indicated a significant association among the conditions, there were several limitations to this descriptive study. We collected nearly the entire population of this DOC (84%), but our mutually exclusive groups for SUD (alcohol, cannabis, intravenous drug, narcotics, and tobacco) and mental health disorders (anxiety, bipolar, depression or depressive symptoms, and psychotic disorders) resulted in small totals for each subgroup and limited complex comparisons, such as controlling for the presence of some variables. While the primary aims of this investigation were to evaluate risks (SUD and mental health disorders) related to suicide attempts in an offender population, we did not have a complete health history related to these conditions. For example, we neither collected the duration and extent of SUD nor the severity of mental health disorders.

With regard to SUD, we did not know the reason for use (i.e., coping, pleasure, or social) that may be explanatory [60]. Information related to treatment history, adherence, or completion (i.e., pharmacological or addiction counseling) was not collected for this investigation, but may be explanatory, particularly in regard to the greater odds for suicide attempts for men compared to women. Men in this DOC may not access mental health services in similar proportions to women. We also did not have a complete history for previous suicidal ideation, attempts, completions, or family history of suicide. Competed suicides were documented in a restricted system and family history, which may be a risk factor [21,27,36,51], was not a routine initial screening question for new arrivals. We also did not have access to clinical notes regarding the details and severity of suicide attempts.

The preliminary findings of this investigation suggest further exploration into SUD, mental health disorders, and co-occurrences. In addition to these conditions, there are a growing number of investigations that have found a relationship between chronic conditions (e.g., diabetes, epilepsy, cardiovascular disease) and self-harm, which was not included or examined as a potential mediating variable [8,61]. Despite several limitations, the majority of the data we did not collect are typically documented. These additional data likely will explain more completely the population at risk for attempting suicide, as well as provide an opportunity for analyses that will be more explanatory than descriptive.

## 5. Conclusions

Corrections is the first and most extensive contact that many offenders have with a health care system. Corrections was mandated by *Estelle v. Gamble* (429 U.S. 97) (U.S. Supreme Court) to provide unfettered access to health care, which includes mental health services and addiction treatment [62]. Thus, offenders with these conditions are provided access to appropriate services, which they may or may not utilize. In regard to SUD (excluding tobacco smoking in some systems), imprisonment largely minimizes use and access to these substances while also providing treatment. However, this DOC did not provide combined and coordinated treatment for the population with co-occurring SUD and mental health disorders, despite having a shared EHR to facilitate coordination and management of care. Clinical notes documenting mental health care were siloed in the EHR. The protection of mental health notes, provided by psychiatrists and psychologists, is understandable, but treatment and interventions for co-occurrences of SUD and mental health disorders are likely more effective when designed and delivered to address both conditions in a coordinated way [4,63,64] while protecting sensitive confidential health information.

The large number of suicide attempts documented during imprisonment compared to the few that occurred prior to prison suggests that corrections recognizes the issues and likely is addressing the problem. The number of suicide attempts that occur during imprisonment is also a call for corrections to continue identifying risk factors, evaluating the correctional environment, and enhancing screenings, especially since prevalence for SUD and mental health disorders changes over time [19]; thus, risk factors, environment, and screenings may also require adjustments. Self-harm in an offender population is not only a corrections issue, but also a public health concern, since the majority of the imprisoned population is released. These condition in released offenders, co-occurring or not, are exacerbated when treatment is unavailable, inaccessible, or too complex to navigate in a non-imprisoned setting. Untreated or poorly addressed SUD, mental health disorders, and co-occurrences that are related to high risk for suicide ideation, attempts, or completion have a large social and economic impact, particularly when individuals become functionally or physically disabled.

Future collaborative investigations between corrections and institutions such as academia, public health, addiction treatment services, and mental health, will be essential for enhancing screening and treatment for groups with these conditions. Treatment programs that were developed for non-imprisoned populations may not be the most effective approach for corrections, which has similarities (but also unique characteristics, socially and structurally) to the general population. The risk factors for self-harm are complex and a more complete identification of characteristics associated with self-injurious thoughts and behaviors by subgroups will enhance screening and triaging into appropriate and complete programs. An assessment of current screening practice may identify unknown gaps that are readily addressable. Longer term investigations to identify risk factors will require larger studies with more complete health information and history. Investigations will need to include more expansive sociodemographic variables, such as marital or family status and measures of social connectedness, as well as the potential effects of physical health conditions. Larger and more comprehensive investigations also will facilitate conducting multivariate analyses to adjust the influences of confounding variables [65]. Data-mining methods, such as random forest, will be important for uncovering and explaining moderation effects associated with factors, such as SUD, mental health disorders, physical health, and self-harm [66].

## Figures and Tables

**Table 1:** Extracted substance use disorders, mental health disorders, and suicide attempt.

ICD-9 Code	ICD-9 Description
**Substance Use Disorders**	
303.9-305	Alcohol dependence, Alcohol abuse
305.9	Intravenous drug user (IDU)
304.2-305.6	Cannabis dependence, Cannabis abuse
304	Heroin dependence
69.8	Narcotic drug user
69.8	Cigarette smoker
305.1	Smoker
**Mental Health Disorders**	
300-300.29	Anxiety, Anxiety disorder, Generalized anxiety disorder, Panic attack, Panic disorder, Panic disorder with agoraphobia, Social phobia
309.81	Posttraumatic stress disorder
296-296.7	Bipolar affective disorder, current episode manic, Bipolar affective disorder, currently depressed, moderate, Bipolar affective disorder, current episode mixed, Bipolar I disorder
296.89	Bipolar II disorder
293.83-296.34	Mood disorder with depressive features due to general medical condition, Depressive disorder, Moderate major depression, Major depressive disorder, recurrent episode, Recurrent major depressive episodes, moderate, Recurrent major depressive episodes, severe, with psychosis
311	Depression
295.3-298.9	Paranoid schizophrenia, Schizophrenia, Psychotic disorder
**Suicide Attempts**	
955.9	Suicide attempt, Suicide attempt by adequate means

ICD-9: International Statistical Classification of Diseases.

**Table 2:** Population demographics.

Variable	*N* (%)
**Race**	
African American	3231 (29.4)
Asian	11 (0.1)
Hispanic	151 (1.4)
Native American	13 (0.1)
Pacific Islander	3 (0)
White	7277 (66.2)
Unknown	302 (2.8)
**Gender**	
Men	9905 (90.1)
Women	1083 (9.9)
**Education**	
Primary	602 (5.5)
Some secondary	1821 (16.6)
High school	2061 (18.8)
Post-secondary	520 (4.7)
Unreported	5984 (54.5)
**Criminal Offense**	
Drug	825 (7.5)
Other	363 (3.3)
Property	2150 (19.6)
Violent	3408 (31)
Unavailable	4242 (38.6)
**Security Level**	
Minimum	1154 (10.5)
Medium	4790 (43.6)
Maximum	1570 (14.3)
Unavailable	3474 (31.6)
**Total**	10,988

**Table 3:** History of substance use disorders, mental health disorders, and suicide attempt.

Variable	*N* (%)	Diagnosis Made	
		Prior to Prison	During Imprisonment
		*N* (%)	*N* (%)
**Substance Use Disorders**			
Alcohol	652 (5.9)		
IDU	285 (2.6)		
Cannabis	747 (6.8)		
Narcotics, all	210 (1.9)		
Cocaine	165 (1.5)		
Heroin	14 (0.1)		
Tobacco smoking	2351 (21.4)		
**Mental Health Disorders**			
Anxiety	972 (8.9)	43 (4.3)	964 (95.7)
Bipolar	277 (2.5)	20 (5.3)	355 (94.7)
Depression/Depressive symptoms	1098 (10)	63 (4.7)	1279 (95.3)
Psychotic	315 (2.9)	32 (6.5)	457 (93.5)
Bipolar and Anxiety	95 (0.9)		
**Suicide Attempts**			
Suicide attempts	378 (3.4)	21 (7.2)	269 (92.8)

**Table 4:** Group differences for suicide attempt.

Variable	No History Suicide Attempt	History Suicide Attempt	χ^2^	*p*	OR	CI
**Race**						
African American	3145 (97.3)	86 (2.7)	10.1	0.001	1.5	(1.2, 1.9)
White (reference)	6993 (96.1)	284 (3.9)				
Total	10,138 (96.5)	370 (3.5)				
**Gender**						
Women	1082 (99.9)	1 (0.1)	40.5	<0.0001	42.8	(6.0, 305.0)
Men (reference)	9528 (96.2)	377 (3.8)				
Total	10,610 (96.6)	378 (3.4)				
**Education**						
Primary	574 (95.4)	28 (4.7)	6.7	0.08	-	-
Some secondary	1767 (97.0)	54 (3.0)				
High school	1999 (97.0)	62 (3.0)				
Post-secondary	509 (97.9)	11 (2.1)				
Total	4849 (96.9)	155 (3.1)				
**Security Level**						
Minimum	1136 (98.4)	18 (1.6)	72.2	<0.0001	-	-
Medium	4628 (96.6)	162 (3.4)				
Maximum	1453 (92.6)	117 (7.5)				
Total	7217 (96.1)	297 (4.0)				
**Substance Use Disorders**						
No SUD history	4263 (97.8)	96 (2.2)	33.3	<0.0001	2.0	(1.6, 2.5)
SUD (reference)	6347 (95.8)	282 (4.3)				
Total	10,610 (96.6)	378 (3.4)				
**Mental Health Disorders**						
No Mental Health Disorder	7214 (99.0)	71 (1.0)	395.6	<0.0001	9.2	(7.1, 11.9)
Mental Health Disorder (reference)	3396 (91.7)	307 (8.3)				
Total	10,610 (96.6)	378 (3.4)				

**Table 5:** Percent suicide attempt by substance use and mental health disorders.

Substance Use/Mental Health Disorder Group	No History Suicide Attempt	History Suicide Related	χ^2^	*p*	OR	CI
**Substance Use Disorders**						
No history of alcohol	9973 (96.5)	363 (3.5)	2.7	0.10	0.6	(0.4, 1.1)
Alcohol use disorder	637 (97.7)	15 (2.3)				
No history of cannabis	9885 (96.5)	356 (3.5)	0.6	0.44	0.8	(0.5, 1.3)
Cannabis use disorder	725 (97.1)	22 (3.0)				
No history of IDU	10,345 (96.7)	358 (3.3)	11.3	0.001	2.2	(1.4, 3.5)
IDU	265 (93.0)	20 (7.0)				
No history of Narcotics	10,405 (96.5)	373 (3.5)	0.7	0.40	0.7	(0.3, 1.7)
Narcotic use disorder	205 (97.6)	5 (2.4)				
No history of tobacco smoking	8392 (97.2)	245 (2.8)	44.3	<0.0001	2.1	(1.7, 2.5)
Tobacco smoking	2218 (94.3)	133 (5.7)				
Total	10,610 (96.6)	378 (3.4)				
**Mental Health Disorders**						
No Anxiety	9680 (96.7)	336 (3.4)	2.5	0.11	1.3	(0.9, 1.8)
Anxiety disorder	930 (95.7)	42 (4.3)				
No Bipolar	10,353 (96.7)	358 (3.3)	12.2	0.001	2.3	(1.4, 3.6)
Bipolar disorder	257 (92.8)	20 (7.2)				
No Depression	9576 (96.8)	314 (3.2)	21.0	<0.0001	1.9	(1.4, 2.5)
Depression	1034 (94.2)	64 (5.8)				
No Psychosis	10,310 (96.6)	363 (3.4)	1.7	0.19	1.4	(0.8, 2.4)
Psychotic disorder	300 (95.2)	15 (4.8)				
No Bipolar with Anxiety	10,530 (96.7)	363 (3.3)	44.0	<0.0001	5.4	(3.1, 9.5)
Bipolar with Anxiety	80 (84.2)	15 (15.8)				
Total	10,610 (96.6)	378 (3.4)				

**Table 6:** Within group differences for suicide attempts.

Substance Use/Mental Health Disorder Group	No History Suicide Attempt	History Suicide Attempt	χ^2^	*p*
**Substance Use Disorders**			70.6	<0.0001
None	4263 (97.8)	96 (2.2)		
Alcohol	637 (97.7)	15 (2.3)		
Cannabis	725 (97.1)	22 (3.0)		
Narcotics	200 (97.6)	5 (2.4)		
IDU	265 (93.0)	20 (7.0)		
Tobacco Smoking	2218 (94.3)	133 (5.7)		
Total	8308 (96.6)	291 (3.4)		
**Mental Health Disorders**			254.9	<0.0001
None	7214 (99.0)	71 (1.0)		
Anxiety	930 (95.7)	42 (4.3)		
Bipolar	257 (92.8)	20 (7.2)		
Depression	1034 (94.2)	64 (5.8)		
Psychotic	300 (95.2)	15 (4.8)		
Bipolar with Anxiety	80 (84.2)	15 (15.8)		
Total	9815 (97.7)	227 (2.3)		

**Table 7:** *p*-Value adjustment for multiple comparisons.

Pairwise Comparisons	Raw	*p*-Value Adjustments		
		Bonferroni	Hommel	Fisher Combination
**Substance Use Disorders**				
No SUD vs. Alcohol	0.89	1.0	0.89	0.95
No SUD vs. Cannabis	0.23	1.0	0.70	0.73
No SUD vs. Narcotics	0.81	1.0	0.89	0.95
No SUD vs. IDU	<0.0001	<0.0001	<0.0001	0.001
No SUD vs. Tobacco smoking	<0.0001	<0.0001	<0.0001	<0.0001
Alcohol vs. Cannabis	0.51	1.0	1.0	0.94
Alcohol vs. Narcotics	1.0	1.0	1.0	1.0
Alcohol vs. IDU	0.001	0.01	0.01	0.06
Alcohol vs. Tobacco smoking	0.0002	0.002	0.002	0.02
Cannabis vs. Narcotics	0.82	1.0	1.0	0.98
Cannabis vs. IDU	0.005	0.05	0.03	0.15
Cannabis vs. Tobacco smoking	0.003	0.03	0.02	0.11
Narcotics vs. IDU	0.02	0.23	0.14	0.33
Narcotics vs. Tobacco smoking	0.05	0.52	0.26	0.47
IDU vs. Tobacco smoking	0.35	1.0	1.0	0.87
**Mental Health Disorders**				
No Mental Health Disorder vs. Anxiety	<0.0001	<0.0001	<0.0001	<0.0001
No Mental Health Disorder vs. Bipolar	<0.0001	<0.0001	<0.0001	<0.0001
No Mental Health Disorder vs. Depression	<0.0001	<0.0001	<0.0001	<0.0001
No Mental Health Disorder vs. Psychotic	<0.0001	<0.0001	<0.0001	<0.0001
No Mental Health Disorder vs. Bipolar with Anxiety	<0.0001	<0.0001	<0.0001	<0.0001
Bipolar vs. Anxiety	0.06	0.59	0.36	0.33
Anxiety vs. Depression	0.13	1.0	0.56	0.48
Anxiety vs. Psychotic	0.75	1.0	0.75	0.80
Anxiety vs. Bipolar with Anxiety	<0.0001	0.001	0.001	0.004
Bipolar vs. Depression	0.40	1.0	0.75	0.74
Bipolar vs. Psychotic	0.22	1.0	0.75	0.59
Bipolar vs. Bipolar with Anxiety	0.02	0.23	0.16	0.20
Depression vs. Psychotic	0.58	1.0	0.75	0.80
Depression vs. Bipolar with Anxiety	0.001	0.01	0.01	0.02
Psychotic vs. Bipolar with Anxiety	0.001	0.01	0.01	0.03

**Table 8:** Odds of suicide attempt for co-occurrence of substance use and mental health disorders.

Substance Use Disorders	Co-occurring Mental Health Disorders	No History Suicide Attempt	History Suicide Attempt	χ^2^	*p*	OR	CI
Alcohol	Depression						
	No history	565 (98.4)	9 (1.6)	11.5	0.001	5.2	(1.8, 15.1)
	Disorder (reference)	72 (92.3)	6 (7.7)				
	Total	637 (97.7)	15 (2.3)				
Cannabis	Depression						
	No history	681 (97.7)	16 (2.3)	15.4	<0.0001	5.8	(2.2, 15.6)
	Disorder (reference)	44 (88.0)	6 (12.0)				
	Total	725 (97.1)	22 (3.0)				
Tobacco smoking	Anxiety						
	No history	2012 (94.6)	116 (5.5)	1.8	0.18	1.4	(0.8, 2.4)
	Disorder (reference)	206 (92.4)	17 (7.6)				
	Total	2218 (94.3)	133 (5.7)				
	Bipolar						
	No history	2151 (94.5)	126 (5.5)	2.1	0.15	1.8	(0.8, 4.0)
	Disorder (reference)	67 (90.5)	7 (9.5)				
	Total	2218 (94.3)	133 (5.7)				
	Depression						
	No history	1964 (94.7)	110 (5.3)	4.1	0.04	1.6	(1.0, 2.6)
	Disorder (reference)	254 (91.7)	23 (8.3)				
	Total	2218 (94.3)	133 (5.7)				
	Psychotic						
	No history	2155 (94.4)	128 (5.6)	0.4	0.54	1.3	(0.5, 3.4)
	Disorder (reference)	63 (92.7)	5 (7.4)				
	Total	2218 (94.3)	133 (5.7)				
